# Loss of epithelium-specific GPx2 results in aberrant cell fate decisions during intestinal differentiation

**DOI:** 10.18632/oncotarget.22640

**Published:** 2017-11-23

**Authors:** Claudia Lennicke, Jette Rahn, Claudia Wickenhauser, Rudolf Lichtenfels, Andreas S. Müller, Ludger A. Wessjohann, Anna P. Kipp, Barbara Seliger

**Affiliations:** ^1^ Institute of Medical Immunology, Martin Luther University Halle-Wittenberg, 06112 Halle (Saale), Germany; ^2^ Institute of Pathology, Martin Luther University Halle-Wittenberg, 06112 Halle (Saale), Germany; ^3^ Delacon Biotechnik GmbH, 4221 Steyregg, Austria; ^4^ Department of Bioorganic Chemistry, Leibniz-Institute of Plant Biochemistry, 06120 Halle (Saale), Germany; ^5^ Institute of Nutrition, Friedrich Schiller University Jena, 07743 Jena, Germany

**Keywords:** glutathione peroxidase 2, selenium, DIGE, stem cells, Clca1

## Abstract

The selenoprotein glutathione peroxidase 2 (GPx2) is expressed in the epithelium of the gastrointestinal tract, where it is thought to be involved in maintaining mucosal homeostasis. To gain novel insights into the role of GPx2, proteomic profiles of colonic tissues either derived from wild type (WT) or GPx2 knockout (KO) mice, maintained under selenium (Se) deficiency or adequate Se supplementation conditions were established and analyzed. Amongst the panel of differentially expressed proteins, the calcium-activated chloride channel regulator 1 (CLCA1) was significantly down-regulated in GPx2 KO versus WT mice regardless of the given Se status. Moreover, transcript levels of the isoforms CLCA2 and CLCA3 showed a similar expression pattern. In the intestine, CLCA1 is usually restricted to mucin-producing goblet cells. However, although -SeKO mice had the highest numbers of goblet cells as confirmed by significantly enhanced mRNA expression levels of the goblet cell marker mucin-2, the observed expression pattern suggests that GPx2 KO goblet cells might be limited in synthesizing CLCA1. Furthermore, transcript levels of differentiation markers such as chromogranin-1 (Chga) for enteroendocrine cells and leucine-rich repeat-containing G-protein coupled receptor 5 (Lgr5) for stem cells were also downregulated in GPx2 KO mice. Moreover, this was accompanied by a downregulation of the mRNA expression levels of the intestinal hormones glucagon-like peptide 1 (Glp1), ghrelin (Ghrl) and somatostatin (Sst). Thus, it seems that GPx2 might be important for the modulation of cell fate decisions in the murine intestinal epithelium.

## INTRODUCTION

Selenium is an essential trace element and modulates via functional selenoproteins a broad spectrum of key biological processes including immune responses, cellular differentiation, redox regulation and maintenance of cellular redox homeostasis [1–3]. For decades Se has been discussed to have beneficial effects in the prevention of different cancer types, including colorectal carcinoma (CRC) [4–6]. The surface of the mammalian gastrointestinal tract (GIT) is self-renewed every few days and is one of the highest proliferative tissues in the organism [7]. The rapid regeneration of the GIT surface is fueled by the proliferation of stem cells located at the intestinal crypt base and their upward migration and differentiation. The differentiated intestinal epithelial cells could be classified as goblet cells, paneth cells, enteroendocrine cells, and tuff cells, which all are derived from the secretory lineage or enterocytes derived from the absorptive lineage. Whereas enterocytes are adapted for metabolic and digestive functions, the other cell types are specialized for maintaining the barrier function of the epithelium and supporting innate immunity. In addition, the hormone-secreting enteroendocrine cells represent a link between the neuroendocrine system and various hormone regulators of the digestive function [8–10]. The signaling pathways Wnt/β-catenin, Notch, bone morphogenic protein (BMP), and epidermal growth factor (EGF) are involved in the control of continuous proliferation and differentiation of the intestine crypt cell populations [11]. Inhibition of the Notch pathway regulates the enterocyte-secretory cell switch and mediates the differentiation into the absorptive cell lineage [12].

The selenoprotein glutathione peroxidase 2 (GPx2) first identified as an epithelium-specific enzyme of the GIT maintains the redox homeostasis by detoxifying hydrogen peroxides (H_2_O_2_) as shown in a CaCo2 cell culture model [13]. *In vitro* studies suggest that GPx2 exhibits anti-inflammatory properties and inhibits the migration of tumor cells, but also supports the growth of transformed intestinal cells [14]. This is in line with the observation that GPx2 is not only expressed in the GIT, but also in various tumor cells of epithelial origin, in the premalignant Barrett´s esophagus leading to esophageal adenomas, in colorectal adenomas and CRCs [15–17]. The expression of GPx2 can be regulated by several transcription factors (TF) which have been also shown to be involved in the mediation of proliferation and differentiation, such as the nuclear factor (erythroid-derived 2)-like 2 (Nrf2), homeobox protein Nkx3.1, β-catenin/TCF, delta-np63 and the STAT family [18–21].

Using a mouse model of inflammation triggered carcinogenesis (azoxymethane (AOM) and dextran sodium sulfate (DSS)) it could be shown that GPx2 knockout (KO) mice developed more tumors, which was also correlated with a more severe DSS-mediated colitis [22]. However, in a mouse model mimicking sporadic CRC (AOM only) GPx2 KO mice developed fewer preneoplastic lesions than WT mice under both Se-deficient and Se-supplemented conditions [23]. Thus, these data suggest an important role of GPx2 in carcinogenesis, which substantially differs depending on the contribution of inflammatory processes to carcinogenesis.

Given that GPx2 is mainly localized at crypt bases, where stem cells are located and that the absence of GPx2 is associated with enhanced apoptosis of crypt epithelia cells [24] the aim of the current study was to identify new candidate proteins, which might be regulated by GPx2 and/or the Se status. Therefore, colonic proteome profiles of GPx2 KO and WT mice fed with either Se deficient or Se adequate diets were analyzed by applying the 2D-difference gel electrophoresis (DIGE) technique. Our data indicate that GPx2 modulates the expression of several proteins involved in differentiation and proliferation processes of this tissue type, in particular CLCA1 and Pax4, leading to an altered distribution pattern of secretory cell types in the colon.

## RESULTS

### Redistribution of selenium into other selenoproteins upon loss of GPx2

**Figure 1:**
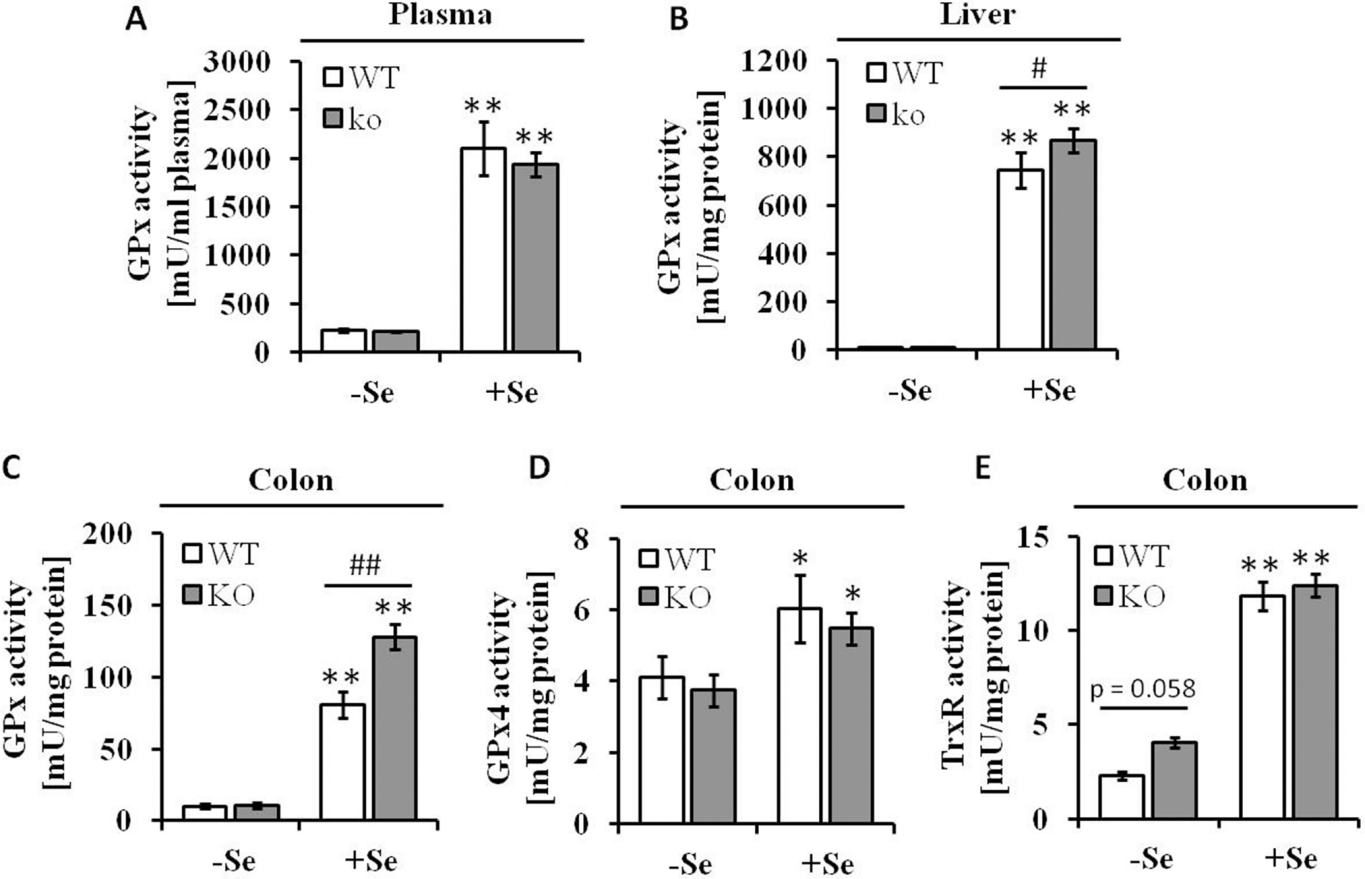
Se status following eight weeks of feeding with different Se concentrations Enzyme activities of (**A**) GPx in plasma, (**B**) GPx in liver and (**C**) in colon lysates, (**D**) GPx4 and (**E**) TrxR in colon tissues were spectrophotometrically determined as described in the Material and Methods section. Values are given as means ± S.E.M. (*n* = 9). Significant differences were calculated by one-way ANOVA. ^*^p < 0.05; ^**^p < 0.01 vs. the respective -Se group; ^##^*p* < 0.01 WT vs. KO within the same Se supply level.

Western blot analysis targeting the GPx2 protein abundance confirmed the GPx2 KO, while a ∼3-fold increased GPx2 protein expression level was detected in the colon of +SeWT mice compared to the corresponding -SeWT mice (Supplementary Figure 1). In line with these data both the systemic selenium status as indicated by defining the plasma GPx activities (Figure 1A) along with the hepatic GPx activity (Figure 1B) and the local Se status in the colon as measured by GPx (Figure 1C) and TrxR activities (Figure 1E) were substantially decreased in the -Se groups. As described before, GPx2 KO mice maintained on the +Se diet exhibited a higher total GPx activity in the colon as well as in liver when compared to the respective WT group. This can be attributed to an increased GPx1 expression upon knockout of GPx2 [24], while GPx4 activity was unaffected by the loss of GPx2 (Figure 1D). In contrast to the GPx activity, total TrxR activity was increased in the GPx2 KO group only under -Se conditions (Figure 1E).

### GPx2 KO alters the proteome profile of colonic tissues of mice

To gain further insights into the function of GPx2 the proteomic profiles of murine colonic tissues from the four experimental groups were compared using the 2D DIGE technology. In total 895 protein spots were detected on the resulting consensus gel (fused image), from which 53 were differentially regulated across the four groups and therefore subjected to MALDI-TOF MS. 19 unique protein IDs could be identified and are listed according to their involvement in cellular processes in Table 1 (see also Supplementary Figure 2): Out of those, six differentially expressed proteins are related to cell differentiation or proliferation, namely acidic leucine-rich nuclear phosphoprotein 32 family member B (AN32B), transgelin-2 (TAGL2), stathmin (STMN1), prelamin A/C (LMNA), heat shock protein HSP 90-beta (HSP84) and calcium-activated chloride channel regulator 1 (CLCA1). The expression of some proteins appeared to be dependent on the given Se status: The dual specificity protein phosphatase 3 (DUSP3) and peroxiredoxin-6 (PRDX6) were downregulated under Se supply, while the protein abundance of the selenoprotein thioredoxin reductase 1 (TR1) [25] was enhanced under these conditions. Furthermore, the GPx2 expression status had an influence on the protein expression pattern of CLCA1 and prelaminin A/C (LMNA9), which were both downregulated in the GPx2 KO groups.

**Table 1:** The GPx2 KO influences the protein expression pattern in colonic tissue of mice

No	protein name	gene			Uniprot ID	mass (kDa)	pI	score	sequence coverage (%)	-Se WT	-Se KO		+Se WT		+Se KO	
	**Cell differentiation and proliferation**															
1	Acidic leucine-rich nuclear phosphoprotein 32 family member B (AN32B)		Anp32b		Q9EST5	31.23	3.89	60	24	1.00	0.60^#^		0.57^*^		0.48	
2	Transgelin-2 (TAGL2)		Tagln2		Q9WVA4	22.55	8.39	58	46	1.00	1.20		0.52^*^		0.53^*^	
3	Stathmin (STMN1)		Stmn1		P54227	17.26	5.76	55	44	1.00	1.07		0.50^*^		0.56^*^	
4	Calcium-activated chloride channel regulator 1 (CLCA1)		Clca1		Q9D7Z6	100.81	5.67	63	22	1.00	0.59^#^		1.34		1.18^*^	
5	Prelamin A/C (LMNA)		Lmna		P48678	74.48	6.54	62	27	1.00	0.75		1.61		1.17^*^	
6	Heat shock protein HSP 90-beta (HSP84)		Hsp90ab1		P11499	83.57	4.97	95	22	1.00	0.87		1.40^*^		1.14	
	**Signal transduction**															
7	Protein kinase C gamma type (PKCγ)		Prkcg		P63318	79.56	7.27	56	16	1.00	1.52^#^		0.68^*^		0.67^*^	
8	Dual specificity protein phosphatase 3 (DUS3)		Dusp3		Q9D7X3	20.69	6.07	64	50	1.00	1.18		0.57^*^		0.56^*^	
9	Calmodulin (CaM)		Calm1		P62204	16.83	4.09	58	53	1.00	0.74		1.27		0.96^#^	
	**Cell metabolism**															
10	Cytochrome c1, heme protein, mitochondrial (CY1)		Cyc1		Q9D0M3	35.53	9.24	72	32	1.00	1.53^#^		0.61		0.61^*^	
11	Cytochrome c1, heme protein, mitochondrial (CY1)		Cyc1		Q9D0M3	35.53	9.24	84	35	1.00	1.34		0.16^*^		0.19^*^	
12	NADH dehydrogenase [ubiquinone] iron-sulfur protein 3, mitochondrial (NDUS3)		Ndufs3		Q9DCT2	30.3	6.67	60	22	1.00	1.22		0.58^*^		0.61^*^	
13	Carbonyl reductase [NADPH] 3 (CBR3)		Cbr3		Q8K354	31.33	6.15	85	39	1.00	1.07		0.53^*^		0.54^*^	
	**Redox homeostasis**															
14	Peroxiredoxin6 (PRDX6)		Prdx6		O08709	24.97	5.71	72	38	1.00		1.36		0.65		0.79^*^
15	Thioredoxin reductase 1, cytoplasmic (TR1)		Txnrd1		Q9JMH6	68.24	7.42	62	22	1.00		1.08		1.51^*^		1.39
	**Others (transport; metal ion binding; cytoskeleton organization)**															
16	Transthyretin (TTHY)			Ttr	P07309	15.88	5.77	61	44	1.00		1.34^#^		0.62^*^		0.71^*^
17	Cytosolic Fe-S cluster assembly factor NUBP2 (NBP 2)			Nubp2	Q9R061	29.90	6.07	58	37	1.00		1.13		0.54^*^		0.58^*^
18	Tropomyosin alpha-1 chain (TPM1)			Tpm1	P58771	32.72	4.69	80	29	1.00		0.93		1.26		1.45^*^
19	Tropomyosin alpha-1 chain (TPM1)			Tpm1	P58771	32.72	4.69	91	34	1.00		1.19		1.37		1.55

Values are given as means (*n =* 4) in relation to the –Se WT group. Levels marked with ^#^ indicate significant differences between WT and KO within the same Se level and levels marked with ^*^ indicate significant differences between WT and KO versus the respective group maintained on –Se conditions. (*p* < 0.05; one-way ANOVA). +Se, selenite supplementation (150 µg Se/kg diet); score, Mascot Probability Based Scoring.

**Figure 2:**
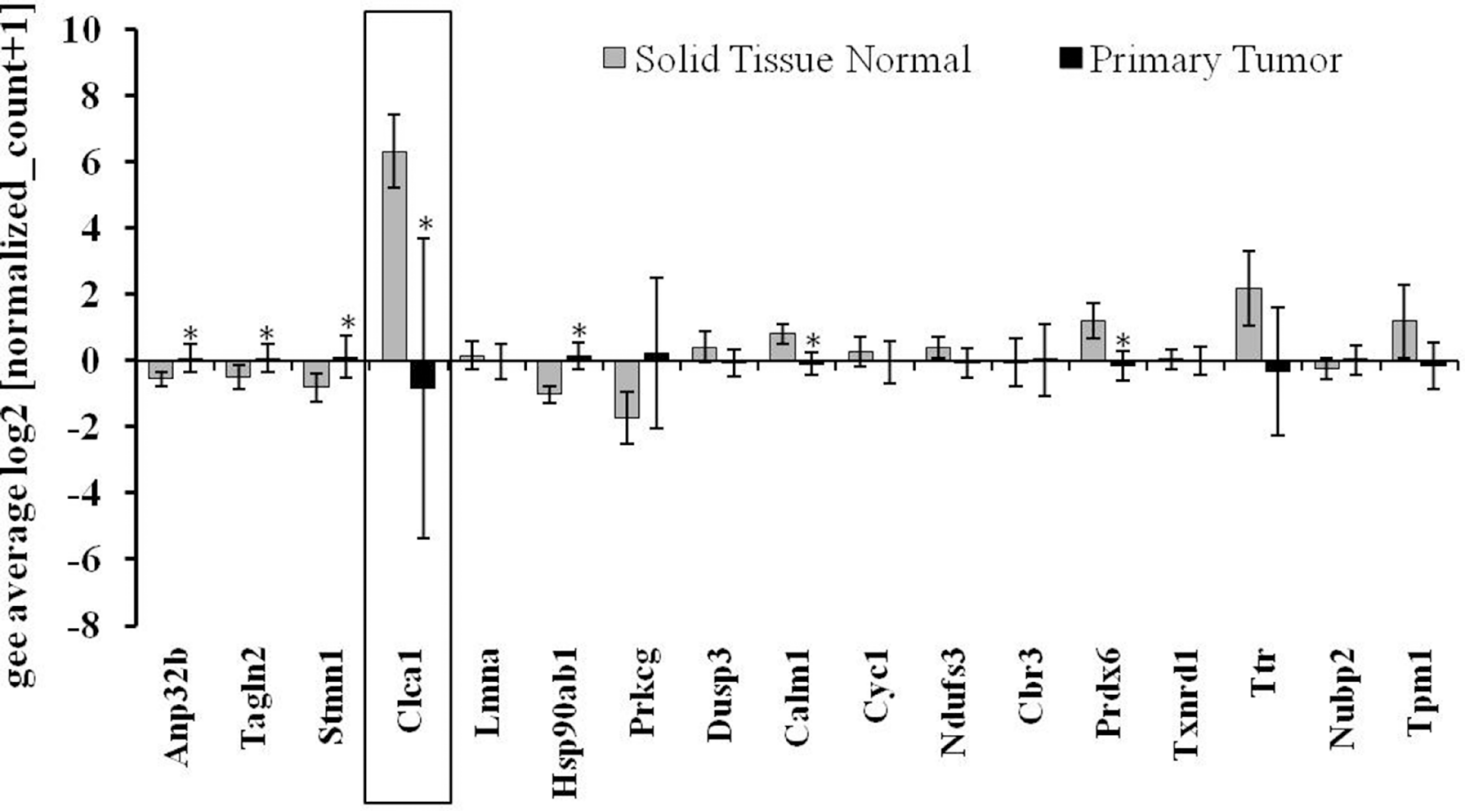
Gene expression pattern of potential targets defined via the murine proteomic profiling experiments in CRC patients Analysis of the expression pattern of proteins, which were found to be differentially regulated by selenium and/or the GPx2 status in the murine model system (see also Table 1) in regard to their potential role in CRC patients (TCGA-COADREAD; *n* = 434). Expression data were Log2 transformed, normalized TCGA-COADREAD data were grouped by the sample types and expressed as means ± S.D. (solid tissue normal *n* = 54, primary tumor *n* = 380). ^*^p < 0.05, student´s *t*-test.

**Figure 3:**
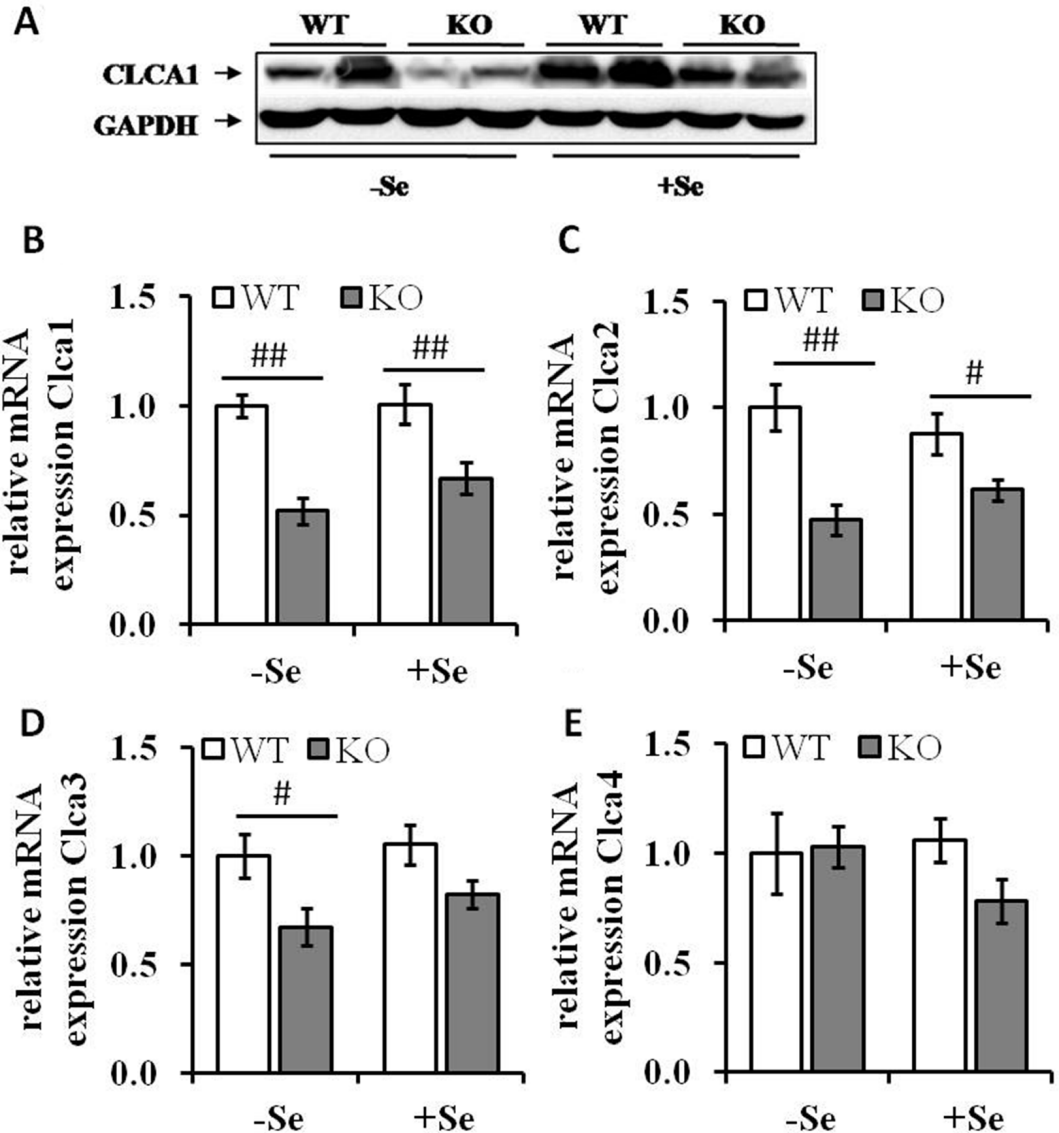
Expression levels of Clca isoforms are decreased by GPx2 knockout (**A**) Protein levels of CLCA1 were analyzed by Western blotting; mRNA expression levels of (**B**) Clca1, (**C**) Clca2, (**D**) Clca3 and (**E**) Clca4 were analyzed by qRT-PCR, normalized to the amplification data of GAPDH, ß-actin and RPL13a and expressed in relation to the –SeWT group. Values are given as means ± S.E.M (*n* = 9 per group). ^#^*p* < 0.05, ^##^*p* < 0.01 (one-way ANOVA).

To identify the most relevant candidates out of the 19 proteins associated with GIT disorders, an *in silico* analysis of their RNA expression levels in human colorectal adenocarcinoma samples was performed (TCGA-COADREAD, *n* = 434). Some of the proteins found to be differentially expressed either by the genotype or by the selenium status were strongly altered in primary tumors compared to normal solid tissues (Figure 2). In particular, Clca1, a protein secreted by goblet cells and known to be involved in the regulation of cell proliferation and differentiation [26] shows a strong down-regulation in tumor samples versus normal tissues. In the proteomic profiling we found decreased expression levels of Clca1 in the two GPx2 KO groups when compared to their respective WT animals (Table 1), which were confirmed at both protein (Western blotting) and mRNA (qPCR) levels (Figure 3A and 3B). Transcription analysis of different Clca isoforms showed that next to Clca1 also Clca2 and Clca3 mRNA levels were decreased in the GPx2 KO groups, whereas Clca4 mRNA expression pattern were not affected (Figure 3C–3E).

**Figure 4:**
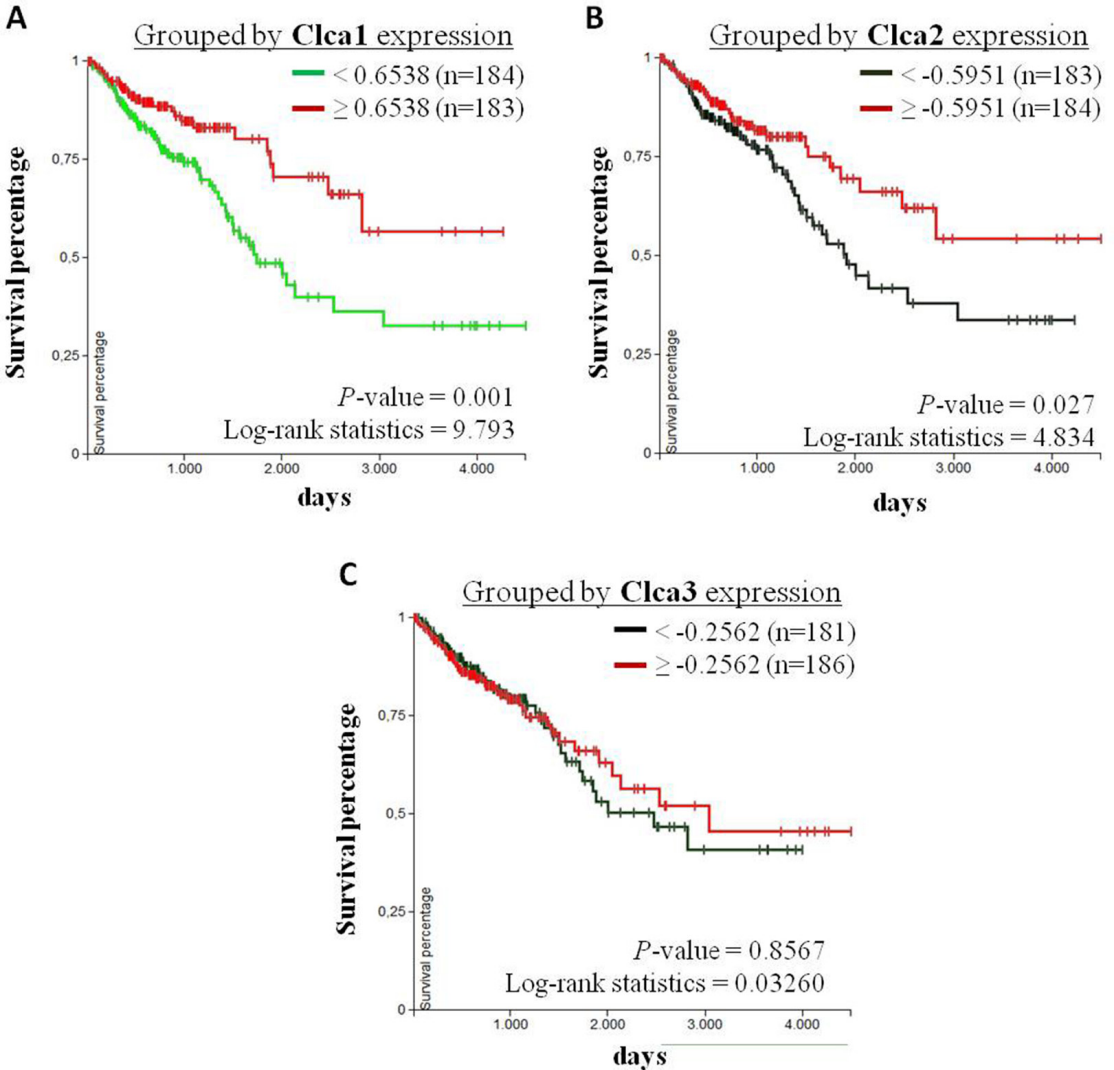
Correlation analysis of Clca isoform expression levels with overall survival of CRC patients Kaplan-Meier curves of CRC patients with primary tumors (TCGA-COADREAD, *n* = 380) were analyzed using the UCSC Xena browser (https://xenabrowser.net). Patients were grouped by the 50 percentiles of (**A**) Clca1, (**B**) Clca2 and (**C**) Clca3 expression levels. The differences between the curves were analyzed with the log-rank test and considered to be statistically significant when *p* < 0.05.

Furthermore, Kaplan-Meier analysis were performed to evaluate the correlation between the survival of CRC patients with primary tumors (TCGA-COADREAD, *n* = 380) and the corresponding expression levels of Clca1, Clca2 and Clca3. Herefore, CRC patients were subdivided into a group with high expression (> 50 %) and a group with low expression (< 50 %) rates of the respective genes. The overall survival (OS) of CRC patients with high Clca1 or Clca2 expression levels is higher than that of patients with low Clca1 or Clca2 expression levels, whereas the expression levels of Clca3 showed no correlation with the patients’ OS rate (Figure 4). However, in this data set no correlation between GPx2 and Clca isoforms was found.

### Increased goblet cell numbers in Se deficient GPx2 KO mice

**Figure 5:**
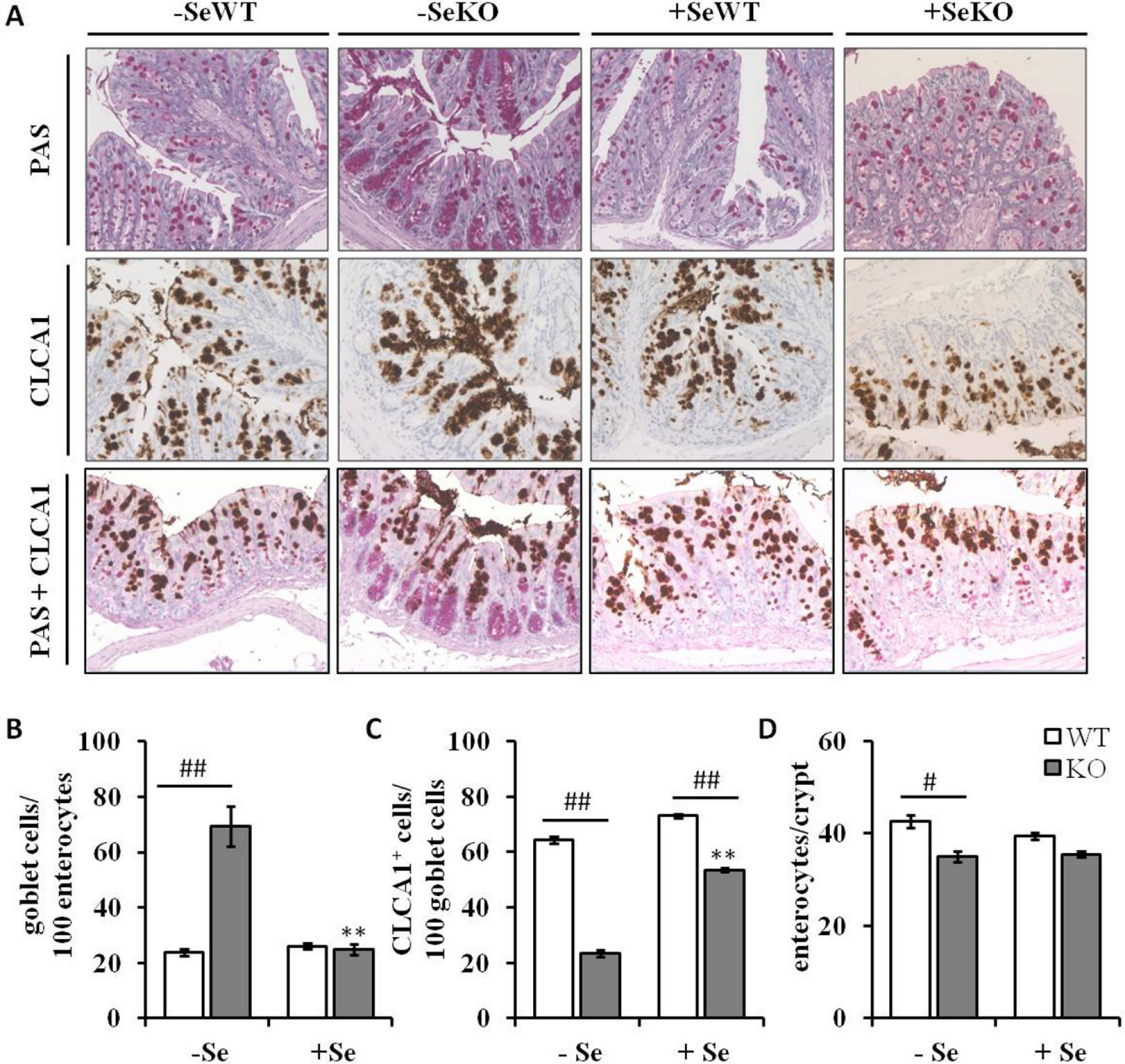
Histological and immunohistochemical analyses of colon sections (**A**) Representative microscopy pictures of colon sections stained with PAS for counting goblet cells (upper row), an anti-CLCA1 antibody for visualizing CLCA1 positive cells (middle row), and co-staining of goblet cells and CLCA1 positive cells (bottom row) (**B**) number of goblet cells per 100 enterocytes, (**C**) number of CLCA1 positive cells per 100 goblet cells and (**D**) number of enterocytes per colon crypt are expressed as means ± S.E.M (*n* = 4 per group). ^*^p < 0.05, ^**^p < 0.01 vs. the respective -Se group; ^#^*p* < 0.05, ^##^*p* < 0.01 WT vs. KO within the same Se supply (one-way ANOVA). HPF, high power field, CLCA1, Calcium-activated chloride channel regulator 1; PAS, periodic acid-Schiff.

Since Clca1 is known to be expressed and secreted by goblet cells in colonic tissues [26], histological analyses were performed to understand whether the decreased Clca1 mRNA and protein expression levels observed within the GPx2 KO groups result from decreased goblet cell numbers. However, whereas GPx2 KO mice maintained on the -Se diet showed increased numbers of PAS-positive goblet cells in comparison to WT mice (Figure 5A, upper row and Figure 5B), no such differences between genotypes were found under +Se conditions. Furthermore, not only goblet cell numbers, but also goblet cell localization within the colonic crypts was changed as a result of GPx2 loss. In -SeWT, +SeWT and +SeKO groups goblet cells were equally distributed throughout whole crypts, whereas in the -SeKO group they accumulated at the crypt base (Figure 5A). These data suggest that the -SeKO epithelium is characterized by an aberrant goblet cell localization (Figure 5A).

### CLCA1 expression does not correlate with goblet cell numbers

Previous studies indicated a co-localization of CLCA1 and mucus-producing goblet cells [26]. Herein, the CLCA1-positive cells do not clearly overlap with goblet cells stained by PAS/AB. In particular, the high number of goblet cells located at the crypt base of the -SeKO group rather lack CLCA1 expression (Figure 5A, lower two rows; Figure 5C). Interestingly, the total number of enterocytes per colonic crypt was solemnly decreased in the –SeKO group (Figure 5D). Taken together this data indicates a dysbalance between the secretory and the absorptive lineage in colon tissues caused by loss of GPx2 in combination with Se deficiency.

### A GPx2 knockout caused an altered expression of differentiation markers of several intestinal cell types

**Figure 6:**
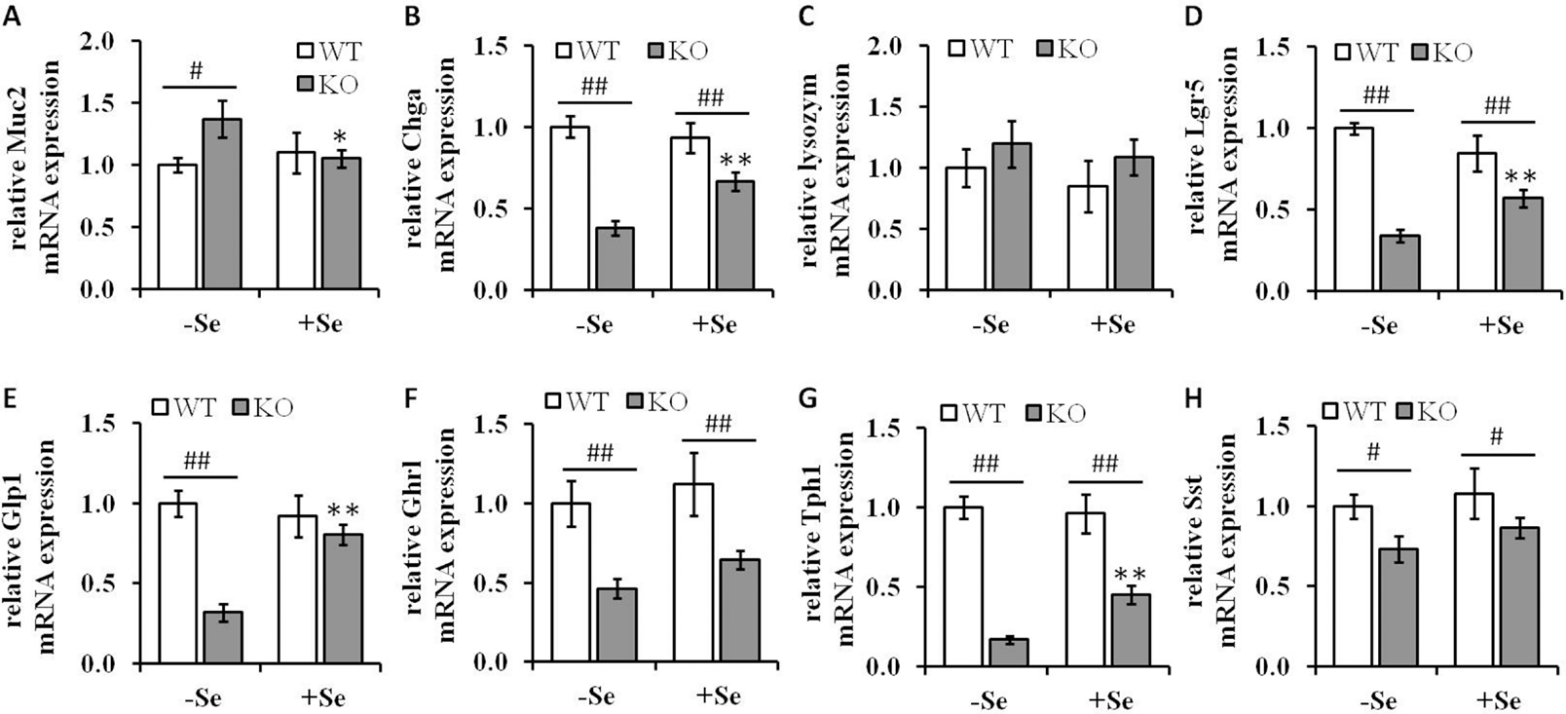
Analysis of differentiation markers in colon lysates of GPx2 KO mice mRNA expression levels of differentiation markers of (**A**) Muc2 (goblet cells), (**B**) Chga (enteroendocrine cells), (**C**) lysozyme (paneth like cells) and (**D**) Lgr5 (stem cells) and the mRNA expression pattern of the intestinal hormones, (**E**) Glp1, (**F**) Ghrl, (**G**) Tph1 and (**H**) Sst were determined by qPCR, normalized to the amplification data of GAPDH, ß-actin and RPL13a and expressed in relation to the -SeWT group. Data are given as means ± S.E.M. (*n* = 9 per group). ^*^p < 0.05, ^**^p < 0.01 vs. the respective -Se group; ^#^*p* < 0.05, ^##^*p* < 0.01 WT vs KO within the same Se supply (one-way ANOVA). Muc2, mucin-2; Chga, chromogranin A; Lgr5, Leucine-rich repeat-containing G-protein coupled receptor 5. Glp1, glucagon-like peptide 1; Ghrl, ghrelin; Tph1, tryptophan 5-hydroxylase 1; Sst, somatostatin.

In order to determine whether the enhanced goblet cell formation observed by the combination of Se deficiency along with the loss of GPx2 affects other cell types, the mRNA levels of cell-type specific differentiation markers were determined (Figure 6). In parallel to the enhanced goblet cell formation, the mRNA expression levels of mucin-2 (Muc2), a glycoprotein synthesized and secreted by goblet cells, were significantly increased in mice of the -SeKO group when compared to all other groups, in which no differences regarding the Muc2 expression levels were found (Figure 6A). In addition, the GPx2 KO significantly altered the expression pattern of markers associated with several cell types present in colon tissues, including chromogranin-A (Chga) (enteroendocrine cells) and leucine-rich repeat-containing G-protein coupled receptor 5 (Lgr5) (stem cells). The mRNA levels of Chga and Lgr5 were clearly significantly downregulated in both GPx2 KO groups when compared to the respective WT groups (Figure 6B and 6D). GPx2 KO mice maintained on the +Se diet exhibited significantly higher expression levels of both markers when compared to the -Se GPx2 KO group. In contrast, the expression levels of lysozyme (paneth cells) were neither affected by the GPx2 genotype nor by the given Se status (Figure 6C).

Since the loss of GPx2 is associated with decreased Chga expression levels, a common marker for enteroendocrine cells, we asked whether this might be associated with altered expression levels of intestinal hormones. Therefore, the mRNA expression levels of the intestinal hormones glucagon-like peptide 1 (Glp1), ghrelin (Ghrl), tryptophan 5-hydroxylase 1 **(**Tph1), and somatostatin (Sst) were analyzed. Glp1 expression was solely down-regulated in the GPx2 KO group maintained on the -Se diet, whereas all the other analyzed hormones showed decreased expression levels in the GPx2 KO groups when compared to the corresponding WT groups (Figure 6E–6H). Intestinal hormone producing cells display a connection between the neuroendocrine and the digestive system and play key roles in regulation of food intake, energy expenditure, glucose and lipid metabolism [27]. In the current study, GPx2 KO mice exhibited altered expression patterns of intestinal hormones and slightly reduced weight gains over the eight weeks feeding period (Supplementary Figure 3).

### Transcription factors involved in modulating differentiation processes

**Figure 7:**
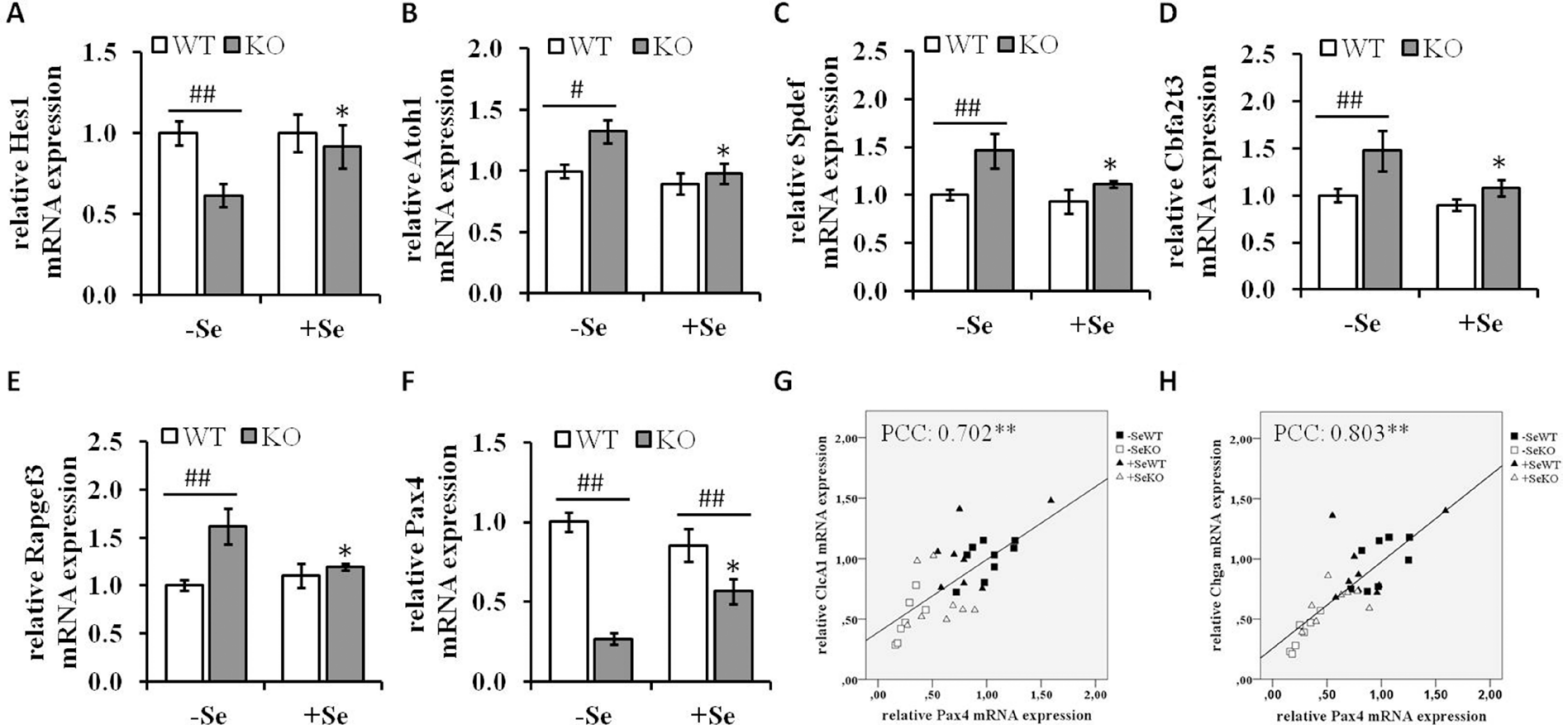
mRNA expression of components involved in differentiation processes mRNA expression levels of the Notch signaling components (**A**) Hes1, (**B**) Atoh1, the Atoh1 downstream targets (**C**) Spdef, (**D**) Cbfa2t3, (**E**) Rapgef3 as well as of the transcription factor (**F**) Pax4 were determined by qRT-PCR normalized to the amplification data of GAPDH, ß-actin and RPL13a and expressed in relation to the –SeWT group. Data are expressed as means ± S.E.M. (*n* = 9 per group). ^*^p < 0.05, ^**^p < 0.01 vs. the respective -Se group; ^#^*p* < 0.05, ^##^*p* < 0.01 WT vs KO within the same Se supply (one-way ANOVA). Correlation analysis of the expression pattern of Pax4, (**G**) Clca1 and (**H**) Chga were conducted by determining the Pearson Correlation Coefficients (PCC). ^**^indicate significant correlations with a *p*-value < 0.01. Atoh1, protein atonal homolog 1; CLCA1, calcium-activated chloride channel regulator 1; Hes1, hairy and enhancer of split-1; Pax4, paired box protein Pax-4.

To gain further insights into the underlying mechanisms leading to the increased goblet cell formation in the -SeKO group, the mRNA expression pattern of important mediators of signaling pathways involved in differentiation processes were analyzed. The Notch pathway regulates the expression of the TF atonal homolog 1 **(**Atoh1), which determines cell fate in the intestine. The transcription factor hairy and enhancer of split 1 (Hes1), a key target of Notch signaling, is important for the differentiation of intestinal absorptive cells, whereas Atoh1 plays a reciprocal role and positively promotes the secretory lineage differentiation. In addition, Atoh1 is repressed by Hes1. In the present study, GPx2 KO mice maintained on -Se diets showed decreased mRNA expression levels of Hes1, whereas the Atoh1 mRNA levels were upregulated (Figure 7A and 7B) thereby suggesting that the enhanced goblet cell numbers observed in this group might at least to some extent be attributed to an aberrant activity of the Notch signaling pathway. To determine whether the enhanced mRNA expression of Atoh1 directly reflect its activity the mRNA expression levels of several Atoh1 downstream targets were analyzed. As shown in Figure 7C–7E, the mRNA expression levels of SAM pointed domain-containing Ets transcription factor (Spdef), Protein CBFA2T3 and Rap guanine nucleotide exchange factor 3 (Rapgef3), which are described to be upregulated by Atoh1 [28], show the same expression pattern like Atoh1. Thus, these results indicate an enhanced Atoh1 activity under GPX2 KO conditions combined with Se deficiency.

Not only Atoh1, but also the TF paired box protein Pax-4 **(**Pax4) is involved in the differentiation of enteroendocrine cells in the intestine, which has been recently demonstrated using Pax4 deficient mice [29, 30]. The expression of Pax4 was strongly reduced in the GPx2 KO groups when compared to their respective WT groups (Figure 7F). Moreover, Pax4 expression levels positively correlated with the expression levels of Clca1 (Pearson Correlation Coefficients (PCC): 0.702, *p* < 0.01) and Chga (PCC: 8.03, *p* < 0.01) (Figure 7G and 7H).

## DISCUSSION

To gain new insights into the functions of the selenoprotein GPx2, which is mainly located in the epithelium of the GIT, comparative proteomic analyses of colon tissues of mice lacking GPx2 expression and of WT counterparts were performed. In addition to the total loss of GPx2 expression, the selenium status was modulated in order to define if the resulting effects can be solemnly attributed towards the loss of GPx2 or if other selenoproteins such as GPx1 might also be involved. Being well aware that different selenium species in nutrition can cause different effects including Gpx1 expression [31, 32], we concentrated on selenite as the most common controllable selenium source. It has been previously reported that under +Se conditions other selenoproteins are up-regulated, which is merely the case under -Se conditions [24]. Thus, effects only seen in -SeKO mice might have been compensated by other selenoproteins under +SeKO conditions.

Based on the performed proteomic profiling analysis of colonic tissues CLCA1 was identified as a potentially downregulated target in both GPx2 KO groups. In addition, Clca1 mRNA was also strongly down-regulated in CRC lesions in comparison to normal tissue samples (TCGA-COADREAD). Furthermore, the Clca1 expression level seems to be of prognostic value since the OS of CRC patients was significantly improved in patients with high tumor-resident Clca1 expression. Similar results as described in this murine model system were recently reported in the context of studies focusing on CRC patients and thus Clca1 is currently discussed to have tumor-suppressive properties [33–35].

CLCA1 represents a multifunctional protein and has been linked to various diseases with mucus overproduction, including cystic fibrosis, asthma and chronic obstructive disease [34, 36–38]. Common features of these diseases are goblet cell hyperplasia, enhanced mucus production and increased expression levels of Clca1. As CLCA1 is secreted by goblet cells together with Muc2 in the GIT [39, 40], it might be involved in the regulation of mucus production and/or goblet cell formation. However, the histological analysis of colon tissues of -SeKO mice showed a marked increase in goblet cell numbers and an induced Muc2 mRNA expression level, but surprisingly low levels of Clca1 expression. Furthermore, in the GPx2 knockout group fed with the +Se diet the reduced Clca1 expression had no impact at all on the goblet cell formation process. Thus, Clca1 expression levels obviously neither correlate with the total goblet cell number nor with the Muc2 expression level in GPx2 KO mice. In addition, CLCA1 expression was rather restricted to goblet cells located in the upper part of the crypts, whereas the high number of goblet cells located at the crypt base observed in the GPx2 KO group maintained on -Se conditions fully lack CLCA1 expression. The authors thus hypothesize that under -SeKO conditions the observed aberrant localization of goblet cells, defined as being mostly stacked at the crypt base, obviously also suppressed their capability to express CLCA1. These results are moreover in line with a recent study using Clca1^-/-^ mice thereby demonstrating neither the presence of Clca1 positive cells at the crypt bases, nor an effect of Clca1 expression on mucus production [41]. To determine whether GPx2 is involved in the transcriptional regulation of Clca1 or the deregulation of CLCA1 occurs due to an aberrant arrangement of the different colonic cell types needs further investigation.

Cell fate decisions between the absorptive and secretory lineage are under control of the Notch signaling pathway. Depletion of Hes1 a direct target of Notch was associated with goblet cell hyperplasia [42], while Atoh1, which is repressed by Hes1, is required for the differentiation into the secretory lineage [43]. As GPx2 KO mice maintained under Se-deficient conditions showed enhanced expression of Atoh1 along with repressed Hes1 expression levels, the enhanced goblet cell formation and localization might be rather attributed to alterations in the Notch signaling pathway than to the aberrant Clca1 expression. Nevertheless, Clca1 is discussed to exhibit anti-proliferative activities and to be required for the spontaneous differentiation of CaCo-2 cells [44]. This suggests that Clca1 might be involved in the proliferation to differentiation transition (PDT) of CRC, which however could be different in the healthy intestine [44]. In normal intestinal epithelium, PDT is a critical step in self-renewal and alterations of the proliferation and differentiation processes lead to the development of diseases, including cancer. Thus, stem cells within the GIT must be constantly ready to respond to external stimuli to maintain normal homeostasis of the epithelium [45]. In the present murine study, the lack of GPx2 expression was associated with a strong down-regulation of the stem cell marker Lgr5. Usually, GPx2 is located at the crypt base, where stem cells are resident and lack of GPx2 is associated with enhanced apoptotic cell death in this area [24]. Therefore, it can be postulated that the reduced Lgr5 expression is attributed to a diminished number of stem cells. However, the whole intestinal integrity is not severely impaired in GPx2 KO mice arguing against this hypothesis. Vice versa, low levels of Lgr5 could also indicate that the Wnt signaling activity of the crypt niche is partially repressed by loss of GPx2 [8]. Using colonosphere cultures of tumor cells recently revealed that loss of GPx2 is associated with enhanced stem cell formation, highlighting differences between functions of GPx2 in tumor models and non-tumor systems. However, in this study overexpression of GPx2 was associated with enhanced transcription levels of the enteroendocrine cell marker Chga [46], which is in line with the observation reported in this study that lack of GPx2 leads to decreased expression levels of Chga. Furthermore, GPx2 KO mice exhibited decreased expression levels of Pax-4, a transcription factor discussed to be involved in the differentiation of enteroendocrine cells in the intestine [29, 30]. In the present study the GPx2 KO was also associated with decreased expression levels of several intestinal hormones, including, Ghrl, Tph1 and Sst. Yet, in Pax-4 deficient mice, a decreased Sst, Tph1, and Chga expression pattern was found, whereas Ghrl was rather upregulated [30]. Thus, Pax-4 might be partially responsible for the observed GPx2-mediated changes in the expression pattern of the respective hormones, but could be also the result of a general decrease in enteroendocrine cells. As intestinal hormones produced by enteroendocrine cells link the neuroendocrine system and various hormone regulators of the digestive function [9, 10] the trend for reduced weight gains of GPx2 KO mice might be a result of deregulated intestine functions.

## MATERIALS AND METHODS

### Animals and diets

C57BL6/J wild-type (WT) and GPx2 KO mice, generated as C57BL/6J;129SV/J hybrid have been backcrossed to the C57BL/6J background before entering the study [23]. Animals were housed under SPF-conditions with a 12 h dark/light cycle and had free access to food and water. At the age of 4 weeks, the mice were randomly assigned to four groups of 9 animals/group (-SeKO, +SeWT, +SeKO) and received diets containing either adequate levels of Se in form of selenite (+Se, 150 µg/kg diet) or a Se-deficient (-Se) diet for eight weeks. Se contents of the diets were confirmed by ICP-MS (-Se, < 20 µg Se/kg diet; +Se, 165 ± 1.9 µg Se/kg diet) as previously described [31]. The diets were based on torula yeast and Se-deficient wheat [31]. After an eight-week feeding period, mice were decapitated under CO_2_ narcosis; blood was collected via heart punction in heparinized tubes and centrifuged for 15 min at 4°C and 2000 x g. Plasma was stored at -80°C until further analysis. Colon and liver tissue samples were excised, snap frozen in liquid nitrogen, and stored at -80°C until further use.

Animal experiments were performed in compliance with the German animal protection law. The mice were housed and handled in accordance with good animal practice as defined by the Federation of Laboratory Animal Science Associations (FELASA, www.felasa.eu/) and the national animal welfare body (GV-SOLAS, www.gv-solas.de/). The animal welfare committees of the German Institute of Human Nutrition (DIfE) as well as the local authorities (Landesamt für Umwelt, Gesundheit und Verbraucherschutz, Brandenburg, Germany) approved all animal experiments.

### Determination of enzyme activities

For enzymatic assays, lysates of colonic and hepatic tissues were prepared in Tris buffer (100 mM Tris, 300 mM KCl, 0.1 % Triton X-100, pH 7.0, Calbiochem® protease inhibitor cocktail II (Merck Millipore, Darmstadt, Germany)) using a TissueLyzer (2 × 30 sec; 30 Hz; Qiagen, Hilden, Germany), centrifuged (14.000 x g, 30 min, 4°C) and stored at -80°C until further analysis. Total GPx and total TrxR activities were determined as previously described [47]. GPx4 activities were measured according to the measurement of total GPx activity, but by applying the specific substrate phosphatidylcholine hydroperoxide (PCOOH, 1.25 mM) instead of 0.00375 % H_2_O_2_ [24].

### Western blot analysis

For Western blot analysis, 30 µg total protein/lane were separated on 10 % SDS-polyacrylamide gels and transferred onto nitrocellulose membranes as described previously [48]. For immune detection the rabbit anti-GPx2 [49] (dilution 1:2000, incubation at 4°C overnight), rabbit anti-CLCA1 (Abcam #180851) and rabbit anti-GAPDH (Cell signaling technology, #2118, New England Biolabs GmbH, Frankfurt, Germany) were used as primary antibodies. HRP-linked anti-rabbit/anti-mouse antibodies (Cell Signaling Technology, #7074, #7076) were used as secondary antibodies.

### Real-time RT-PCR

RNA was isolated and then subjected to PCR analysis as recently described [31]. Target- specific primers were designed using the program Primer3 [50] and are shown in Supplementary Table 1. Amplification data were analyzed according to the method of Pfaffl [51]. For normalization amplicons of GAPDH, ß-actin and RPL13a were employed.

### Analysis of the protein expression pattern by difference gel electrophoresis (DIGE) and mass spectrometry

Four independent biological replicates per group were used for 2D-DIGE analysis based on the minimal labeling approach according to the manufacturer’s instructions (SERVA Lightning SciDyes, SERVA Electrophoresis, Heidelberg, Germany). 25 µg total protein of each sample was labeled with 200 pmol of either SciDye3- or SciDye5 and the internal protein standard was labeled with 200 pmol of the SciDye2. 2DE separation was performed as recently described [52]. For gel image analyses the Delta2D software package (Decodon GmbH, Greifswald, Germany) was used. Whereas differentially expressed proteins were classified by a fold change ratio of 1.5 (increased or decreased) and a *p-value* of < 0.05, mass spectrometry analyses were performed on an ultrafleXtreme™ matrix-assisted laser desorption/ionization time of flight mass (MALDI-TOF) mass spectrometer (Bruker Daltonics Inc., Bremen, Germany). The resulting peptide mass fingerprinting datasets were analyzed using the MASCOT software package (Matrix Science, Dauheim, USA) [53].

### Histological staining

Colon tissues of mice (*n* = 4/group) were washed with 4% formaldehyde and then fixed overnight in 4 % formaldehyde. Fixed colon samples were embedded in paraffin, cut into 4 µm slices and prepared for periodic acid-Schiff (PAS)/Hematoxylin-eosin (HE) staining according to standard protocols. For immunostaining the rabbit anti-CLCA1 antibody (Abcam #180851) was used as primary antibody followed by HRP-linked polymer system detection. Evaluation of PAS positive goblet cells and evaluation of CLCA1 positive cells were performed by counting the cells in a 0.5 mm^2^ high power field (HPF) [54]. Additionally, the number of PAS-positive cells were normalized to 100 enterocytes.

### *In silico* analysis of TCGA data of CRC patients

The UCSC Xena Cancer Genomics Browser (https://xenabrowser.net/) [55, 56] was used to visualize TCGA genomic data (cohort TCGA Colon and Rectal Cancer (COADREAD), 434 patients; poly-A+illimuina hiSeq). The gene expression data was sorted according to the sample types: solid tissue normal, *n* = 54; primary tumor, *n* = 380. In addition, Kaplan-Meier curves of CRC patients with primary tumors were generated using the same tool by analyzing the 50% percentiles of RNAseq data of the respective genes. The analysis was conducted using the UCSC Xena tool (log Rank test) [55, 56]. Data were considered as significantly different if *p* < 0.05.

### Statistical analysis

Mean values were calculated from 9 animals per group or 4 animals per group in the case of the proteomics and Western blot analysis, respectively, and given as means ± their standard error of the mean (S.E.M). SPSS 20 was used to analyze significant differences within the groups. One-way ANOVA (LSD post hoc test) was applied if the normality of distribution (Shapiro-Wilk test) and the homogeneity of variance (Levene test) were defined. Otherwise, the Games-Howell test was employed. If only two groups were compared the student´s *t-test* was applied. Differences between the groups were considered to be significant at *p* < 0.05.

## CONCLUSIONS

In summary, the current study indicates that loss of GPx2 modulates the expression levels of cell-type specific markers, which might reflect a dysbalance between absorptive and secretory cell types. In addition, not only the number, but also the localization of differentiated cell types was changed upon loss of GPx2. The exact underlying mechanisms still need to be resolved, but might involve the Notch signaling pathway as well as the transcription factor Pax-4. Some of the effects found in the current study only became obvious when the GPx2 KO was combined with Se deficiency conditions. The results indicate that other selenoproteins, most probably GPx1, can compensate at least to some extent for the loss of GPx2 during intestinal differentiation, but only in the presence of sufficient Se. As both GPx1 and GPx2 are major antioxidant enzymes, redox modification of signaling pathways such as Notch and Wnt might be the cause for aberrant cell fate decisions as defined within this report.

## SUPPLEMENTARY MATERIALS FIGURES AND TABLE



## Abbreviations

AOM: azoxymethane
Atoh1: protein atonal homolog 1
Chga: chromogranin A
CLCA: Clca, Calcium-activated chloride channel regulator
CRC: colorectal carcinoma
DIGE: difference gel electrophoresis
DSS: dextran sodium sulfate
GAPDH: glyceraldehyde 3-phosphate dehydrogenase
Ghrl: ghrelin
GIT: gastrointestinal tract
Glp1: glucagon-like peptide 1
GPx: glutathione peroxidase
Hes1: hairy and enhancer of split-1
HPF: high power field
KO: knockout
Lgr5: leucine-rich-repeat-containing G-protein coupled receptor 5
MALDI-TOF MS: Matrix-assisted laser desorption/ionization time of flight mass spectrometry
Muc2: mucin-2
Nrf2: nuclear factor (erythroid-derived 2)-like 2
OS: overall survival
PAS: periodic acid-Schiff
PAX-4: paired box protein
PCC: Pearson Correlation Coefficients
PDT: proliferation to differentiation transition
Se: selenium
Sst: somatostatin
TCGA: The Cancer Genome Atlas
TF: transcription factor
Tph1: tryptophan 5-hydroxylase 1
TrxR: thioredoxin reductases
WT: wild type
