# Supplementary material for: Loss of epithelium-specific GPx2 results in aberrant cell fate decisions during intestinal differentiation
Source: Oncotarget. 2017 Nov 23;9(1):539–52. doi: 10.18632/oncotarget.22640 (PMC5787487; doi:10.18632/oncotarget.22640)
Supplement: Supplementary file 1 [file oncotarget-09-539-s001.pdf]

Loss of epithelium-specific GPx2 results in aberrant cell fate decisions during intestinal differentiation

SUPPLEMENTARY MATERIALS

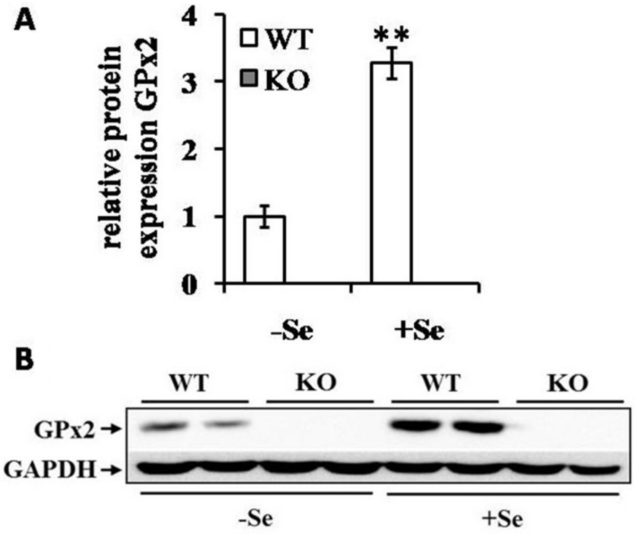

Supplementary Figure 1: Confirmation of the GPx2 KO by western blot analysis. (**A**) Colon tissue lysates of four mice/group were analyzed for GPx2 protein abundance by Western blot, quantified by densitometry, normalized to the expression of GAPDH and expressed in relation to the -SeWT group. $^{**}p < 0.01$, student´s *t*-test. (**B**) Representative Western blots of two animals/group.

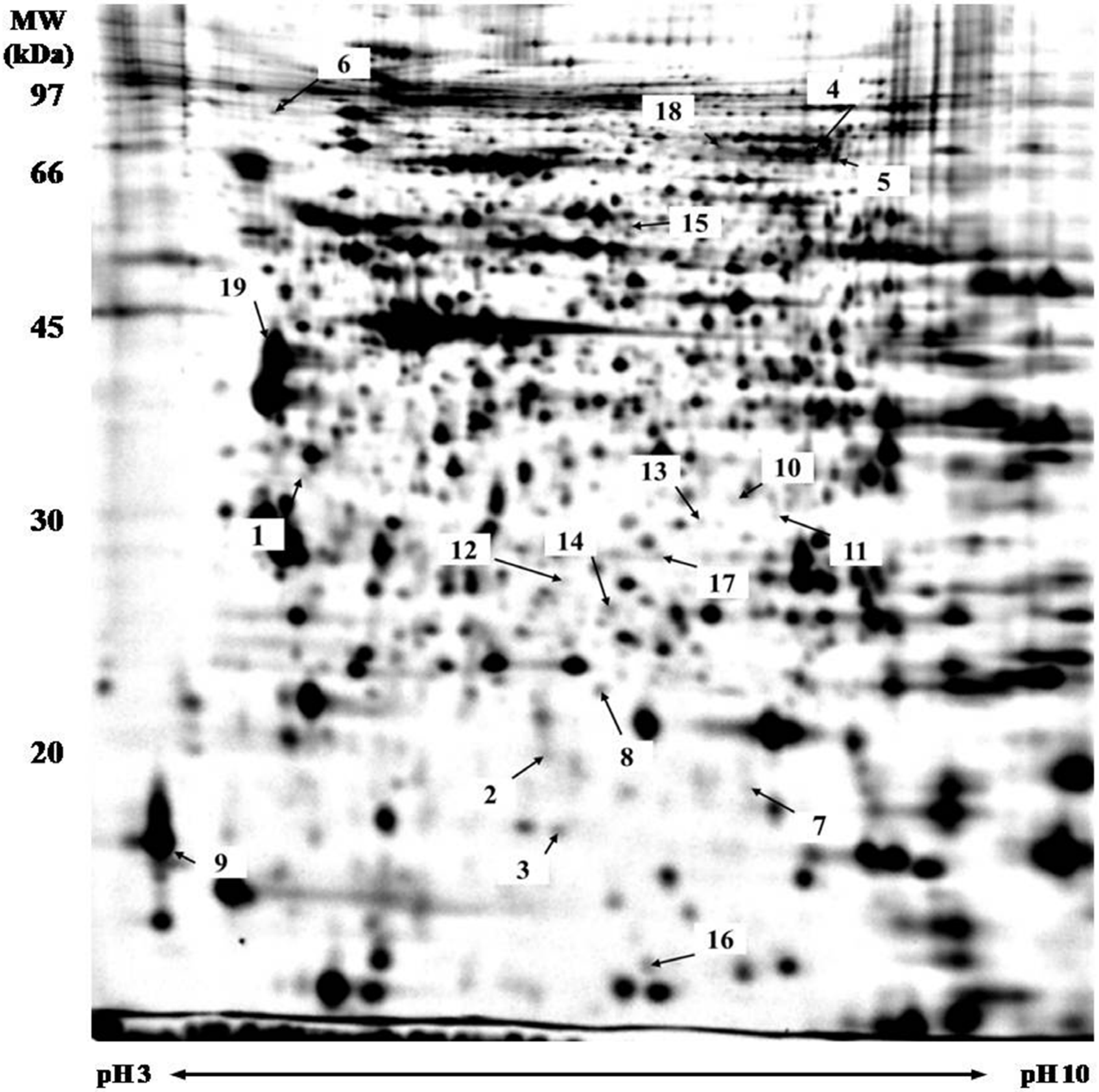

Supplementary Figure 2: GPx2 KO influenced the protein expression pattern of murine colon tissues. Representative proteome profile of colon tissues (sample + Se WT) generated as described in the Materials and Methods section. Significant differentially expressed and identified proteins are indicated with numbers (p < 0.05; see also Table 1).

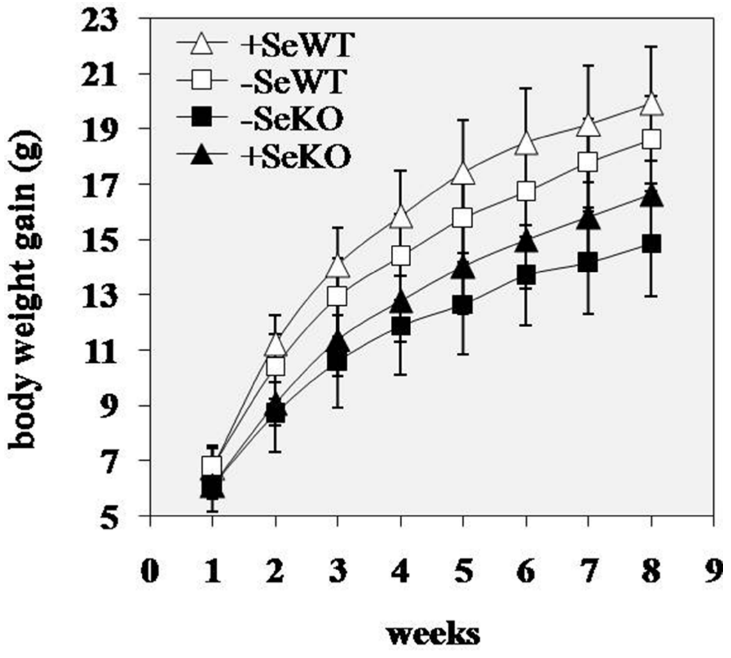

Supplementary Figure 3: GPx2 knockout mice exhibit decreased weight gain. Weights of the mice were recorded weekly and the cumulative weight gain (g) was calculated across the eight-week feeding period.

Supplementary Table 1: Real-time PCR primer pairs applied in this study

gene		primer sequence (5′→3′)	gene bank accession No.	product size (bp)
Atoh1	forward	AGAAGCTGTCCAAATATGAGACCC	NM_007500.4	141
	reverse	GTGCGAAGGTGATGGTGGTC		
Cbfa2t3	forward	TCTGAGGAGGGGAAGAAGGG	NM_177289.1	356
	reverse	CAGTTCCAGCAGCTCTCGC		
Chga	forward	TACCCAATCACCAACCAGCC	NM_007693.2	249
	reverse	GAGTCCGACTGACCATCATCT		
Clca1	forward	GATAACGGAGCAGGTGCCG	NM_017474.2	486
	reverse	TGAGGTGTTGAAGTGGTCCC		
Clca2	forward	GTCACCAGGTGTTCATCTGAC	NM_178697.5	257
	reverse	ACGTCCCAAGTGCTTCTGAG		
Clca3a1	forward	GACCCCGAGGCAGAGTCTTT	NM_009899.4	150
	reverse	CCACATTGGTGCCAGTGATCC		
Clca4a	forward	CAACAAGGTGCTCCACAGG	NM_207208.3	343
	reverse	GAGAAGAAGGGTGGAGAGGG		
Ghrl	forward	CCAGAGGACAGAGGACAAGC	NM_021488.5	170
	reverse	GCTGGCGCCTCTTTGACC		
Glp-1	forward	TGTCTACACCTGTTCGCAGC	NM_008100.4	151
	reverse	AAGCTGGGAATGATCTGGGG		
Hes1	forward	AACACGACACCGGACAAACCA	NM_008233.2	150
	reverse	GCCGGGAGCTATCTTTCTTAAGTG		
Lgr5	forward	AAGTTCAAGATGAGCGGGACCT	NM_010195.2	142
	reverse	GATTCGGATCAGCCAGCTACCA		
Lysozym	forward	TTCGAGCATGGGTGGCATGG	NM_013590.3	100
	reverse	GGCTGCAGTAGAAGCACACC		
Muc2	forward	ACTGTTTTGGGTCCTGTGGG	NM_023566.3	241
	reverse	AGAAAACGGGGGCTAGAACG		
Pax4	forward	CTGCTCCTGAGTGAAGGCTC	NM_011038.2	277
	reverse	TCACACTGCTGAGTCCGTCC		
Rapgef3	forward	CTGCTGCTCAAAGACGTGACC	NM_001177810.1	393
	reverse	CCTCTGGACCTCTTGGTTGC		
Spdef	forward	ACATCTGGAAGTCAGCGGC	NM_013891.4	319
	reverse	TAATACTGGCGGATGGAGCG		
Sst	forward	TGCCACCGGGAAACAGGAAC	NM_009215.1	322
	reverse	GTCAAGTTGAGCATCGGGGG		
Tph1	forward	TACACTCCAGAGCCAGACACC	NM_009414.3	415
	reverse	TGTACTTCAGTCCAAACGGGC		
beta-actin	forward	ACTGTCGAGTCGCGTCC	NM_007393.5	487
	reverse	ATGGCTACGTACATGGCTGG		
Gapdh	forward	TTGTGCAGTGCCAGCCTCGT	NM_008084.3	214
	reverse	TCGGCCTTGACTGTGCCGTT		
Rpl13a	forward	GACCTCCTCCTTTCCCAGG	NM_009438.5	148
	reverse	TTCACAGCGTACGACCACC		
